# Efficacy of deep brain stimulation of the subthalamic nucleus versus globus pallidus internus on sensory complaints

**DOI:** 10.1038/s41531-024-00689-z

**Published:** 2024-03-29

**Authors:** Maria Gabriela S. Ghilardi, Ana Carolina P. Campos, Rubens G. Cury, Raquel C. R. Martinez, Rosana L. Pagano, Erich T. Fonoff

**Affiliations:** 1 Division of Functional Neurosurgery, Department of Neurology, University of São Paulo Medical School, São Paulo, São Paulo Brazil; 2 Laboratory of Neuroscience, Hospital Sírio-Libanês, São Paulo, São Paulo Brazil; 3 Movement Disorders Center, Department of Neurology, School of Medicine, University of São Paulo, São Paulo, São Paulo Brazil; 4 Hospital Israelita Albert Einstein, São Paulo, Brazil; 5 LIM/23, Institute of Psychiatry, University of São Paulo School of Medicine, São Paulo, São Paulo Brazil

**Keywords:** Neurological manifestations, Parkinson's disease

## Abstract

Pain control after deep brain stimulation (DBS) in Parkinson’s disease (PD) remains unclear. Following six months, subthalamic (STN)-DBS reduced sensory complaints related to parkinsonism and bodily discomfort, increasing central beta-endorphin level. Pallidal GPi-DBS decreased bodily discomfort and beta-endorphin levels. Unexplained pain by other conditions and bodily discomfort were negatively correlated with beta-endorphin levels. Thus, DBS regulates central opioids, and prioritizing STN is important for PD patients with significant sensory complications.

## Introduction

Deep brain stimulation (DBS) has become routinely used in the treatment of Parkinson’s disease (PD) due to its proven efficiency in attenuating the motor symptoms of PD^1^. However, the role of different targets of DBS in modulating the non-motor symptoms of PD, especially persistent pain, has yet to be fully comprehended. Subthalamic nucleus (STN) stimulation has been shown to increase the heat pain threshold, correlating with neuroimaging activation in the primary somatosensory cortex and insula of PD patients with painful symptoms^2^. In 2016, a study led by the IPMDS Non-Motor PD Study Group demonstrated in a multicenter, open, prospective study that STN-DBS attenuates the miscellaneous domain of the non-motor symptom scale^3^. Moreover, in a retrospective study, Gong and coworkers found that both globus pallidus internus (GPi) and STN-DBS significantly reduced pain perception evaluated by the numerical rating scale (NRS)^4^. However, in contrast to other studies, Gierthmühlen and coworkers showed that while 4 out of 12 patients who experienced painful symptoms prior to STN-DBS had complete subjective pain relief up to 40 weeks after the surgical procedure, no significant modulation was observed in pain thresholds evaluated by quantitative sensory testing (QST)^5^. Taken together, these data suggest the complexity of pain physiopathology and the limitations of objective assessment of pain in PD. Moreover, recently, our group, in collaboration with the EUROPAR and IPMDS Non-Motor PD Study Group, has shown that only STN-DBS stimulation improves pain, attention, and memory, while both targets improved sleep, fatigue, mood, and cognition^6^.

Persistent pain is one of the major non-motor symptoms related to PD^7,8^. Considering the impact of persistent pain on the quality of life of these patients is pivotal to better comprehend the role of DBS in painful behavior. Furthermore, unraveling the biomolecular effects induced by DBS may explain its efficiency or failure. In this sense, the opioidergic system plays a significant role in pain modulation^9^, as demonstrated in PD preclinical models^10^ and less invasive functional surgeries^11^. This study aimed to understand the range of pain characteristics and central opioidergic system involvement by comparing individuals before and after STN-DBS and GPi-DBS. We evaluated pain parameters in relation to: (i) pain not explained by other known conditions (subscore of NMSS item 27), (ii) sensory complaints related to parkinsonism (subscore of UPDRS-II item 17), (iii) bodily discomfort (subscore of PDQ-39), and the expression of beta-endorphin in the cerebrospinal fluid (CSF), before and six months after DBS implantation.

## Results

### Demographics and clinical data

For that purpose, we have included twenty-seven patients with PD who were randomly subjected to STN-DBS (*N* = 13) or GPi-DBS (*N* = 14). The age (in years) and neurological features are presented in Table 1. There were no differences in terms of age and years since diagnosis. Subthalamic and pallidal stimulation improved total motor score in the UPDRS-III/OFF and overall quality of life in PDQ-39 when comparing baseline versus 6 months after the surgery, while no significant differences were found between different targets. As expected, only STN-DBS significantly decreased the levodopa-equivalent daily dosage (LEDD) and the pramipexole dosage (STN = −0.82 vs. GPi = +0.05). Furthermore, no significant differences were found regarding other features present in the NMSS score, including cardiovascular (STN = 0.07 vs. GPi = 0.33), sleep/fatigue (STN = 8.07 vs. 7.9), and gastrointestinal disturbances (STN = 5.6 vs. GPi = 2.53).

**Table 1:** Neurological features

	STN	GPi	STN vs. GPi
Age, years (median, range)	49.8 (±9.2)	52.7 (±7.8)	0.320
**Neurological features**			
Years since PD diagnosis, years (mean, ±SD)	12.1 (±4.8)	12.3 (±3.5)	0.89
UPDRS-III/ON (points)*			
Baseline	13 (±6.5)	12.07 (±7.1)	0.98
6 months	11 (±7.1)	11.43 (±5.3)	0.99
UPDRS-III/OFF (points)*			
Baseline	44.20 (±7.7)	42.29 (±7.7)	0.93
6 months	22.13 (±9.6)^**b**^	26.43 (±8.9)^**b**^	0.530
PDQ-39 (points)*			
Baseline	49.2 (±11.7)	54.7 (±10.4)	0.650
6 months	28.7 (±13.8)^**b**^	29.5 (±14.8)^**b**^	0.990
Magnitude of pain related to off-dystonia			
Baseline	1.6 (±2.0)	0.67 (±1.1)	0.06
6 months	0.3 (±0.4)^**b**^	0.5 (±0.9)	0.67
LED (mg/24 h)*			
Baseline	1338.2 (±398.7)	1273.1 (±564.1)	0.980
6 months	728.0 (±446.9)^**b**^	995.7 (±561.6)	0.47
**Correlations with UPDRS-III/OFF (6mo - baseline)**			
vs. Sensory complains related to parkinsonism (6mo - baseline)			*r* = 0.16 *p* = 0.43
vs. Pain not explained by other conditions (6mo - baseline)			*r* = 0.004 *p* = 0.98
vs. Bodily discomfort (6mo - baseline)			*r* = -0.008 *p* = 0.96

Number (Percent) or Median (25–75%). Data expressed as medians and percentiles, Mann-Whitney and Spearman correlation tests. *GPi* globus pallidus internus, *SNT* subthalamic nucleus, *PD* Parkinson’s disease, *LED* levodopa equivalent dose; *UPDRS-III OFF* Unified Scale of Parkinson’s Disease part III without medication; *UPDRS-III ON* Unified Scale of Parkinson’s Disease part III with levodopa medication. b: *p* < 0.05 when compared to baseline.

### Deep brain stimulation, pain-related subscores and beta-endorphin expression

**Fig. 1:**
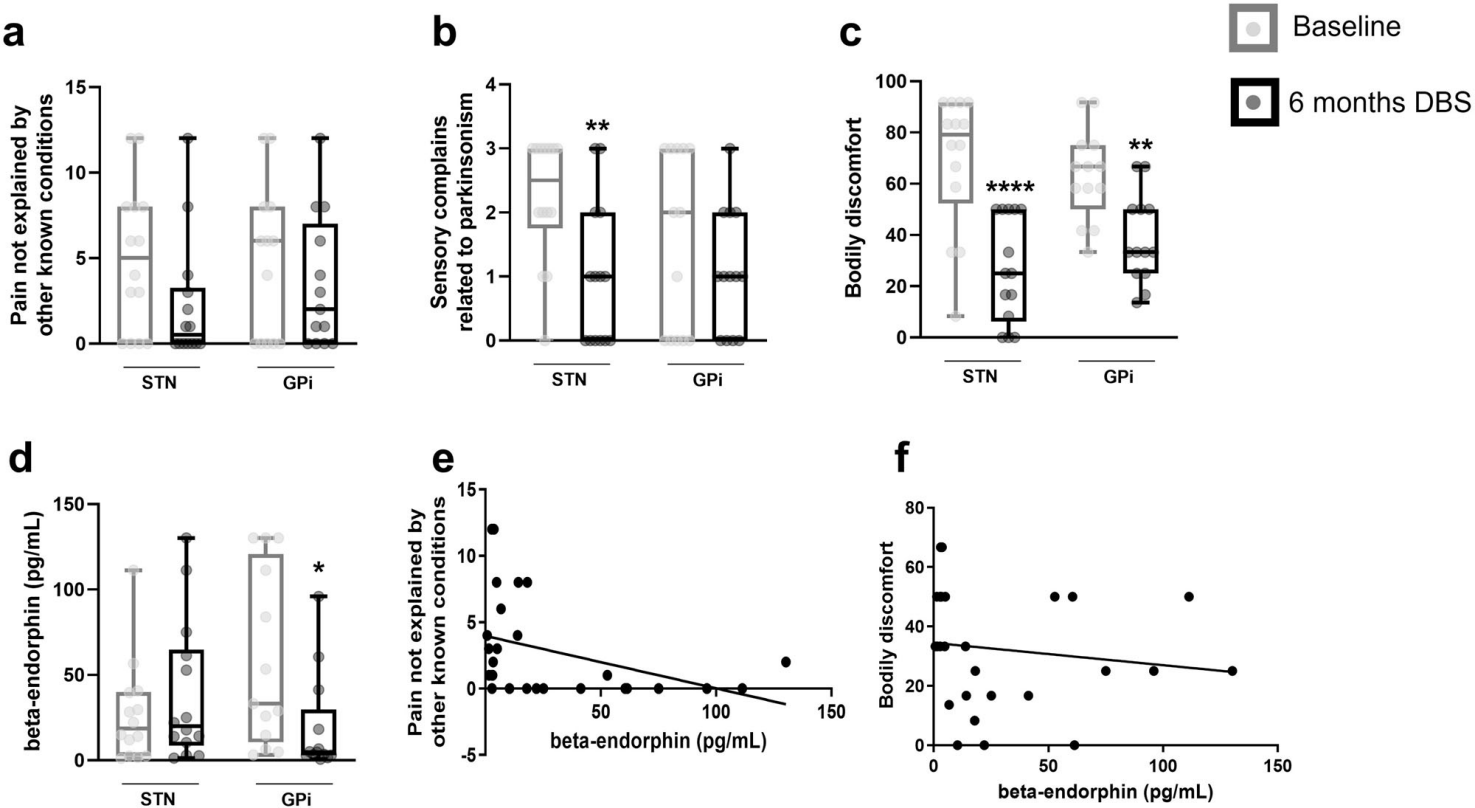
Pain-related subscores and beta-endorphin expression. Clinical evaluation (**a-c**)– of patients submitted to STN-DBS or GPi-DBS before and six months after the surgical procedure. Patients were evaluated for pain not explained by other known conditions (item 27 from the NMSS, **a**), sensory complaints related to parkinsonism (item 17 from the UPDRS-II, **b** and bodily discomfort (item 39 from the PDQ, **c).** Expression of beta-endorphin (**d**) in the cerebrospinal fluid of patients submitted to STN-DBS or GPi-DBS was evaluated before and six months after the surgical procedure. In the boxplots, the centre line represents the median, while upper and lower part of the boxplot represent the 25th and 75th percentiles. Two-tailed Wilcoxon matched-pairs test. *p < 0.05; **p < 0.01; ****p < 0.0001 when compared to pre-surgical values of their respective group. Sensory complications and beta-endorphin correlation (**e** and **f**). The levels of beta-endorphin inversely correlate with pain not explained by other known conditions (*r* = −0.5, *p* = 0.008, Spearman correlation test, **e**), and with bodily discomfort (*r* = −0.4, *p* = 0.041, Spearman correlation test, **f**). DBS Deep brain stimulation, GPi globus pallidus internus, NMSS Non-Motor Symptoms Scale, PDQ Parkinson’s Disease Questionnaire, STN Subthalamic nucleus, UPDRS-II Unified Parkinson’s Disease Rating Scale Part II.

Patients in the STN-DBS group showed a non-significant decrease of 50% in pain not explained by other known conditions (baseline: 5.0 ± 4.3 vs. 6 months after DBS: 2.2 ± 3.6, *p* = 0.084; Fig. 1a). However, a significant decrease in sensory complaints related to parkinsonism was observed (baseline: 2.2 ± 0.9 vs. 6 months after DBS: 1.0 ± 0.5, *p* = 0.007; Fig. 1b), as well as in bodily discomfort (baseline: 69.1 ± 26.4 vs. 6 months after DBS: 26.7 ± 20.4, *p* = 0.001; Fig. 1c). No statistical difference was found in the central beta-endorphin expression evaluated in the CSF (baseline: 27.0 ± 29.6 vs. 6 months after DBS: 38.6 ± 41.6, *p* = 0.426), even though an increase of 43% was observed after stimulation treatment (Fig. 1d).

Patients in the GPi-DBS group showed no statistical difference in pain not explained by other known conditions (baseline: 4.7 ± 4.5 vs. 6 months after DBS: 3.4 ± 3.9, *p* = 0.371; Fig. 1a) and sensory complaints related to parkinsonism (baseline: 1.5 ± 1.4 vs. 6 months after DBS: 1.07 ± 0.9, *p* = 0.222; Fig. 1b). However, a significant decrease was observed in bodily discomfort (baseline: 63.5 ± 17.8 vs. 6 months after DBS: 38.2 ± 17.2, *p* = 0.003; Fig. 1c). Additionally, central beta-endorphin expression was significantly decreased (baseline: 58.2 ± 51.6 vs. 6 months after DBS: 19.05 ± 29.3, *p* = 0.033) after six months of stimulation (Fig. 1d).

No correlation was found between the change (delta) in UPDRS-III/OFF from baseline to 6 months and the pain-related subscores evaluated (Table 1). However, we found that the levels of beta-endorphin evaluated in the CSF of both SNT-DBS and GPi-DBS groups, six months after the stimulation, were negatively correlated with pain not explained by other known conditions (*r* = −0.5, *p* = 0.008; Fig. 1e) and bodily discomfort (*r* = −0.4, *p* = 0.041; Fig. 1f).

## Discussion

As previously discussed, beyond improving the classical motor deficits related to PD, both pallidal and subthalamic stimulation are effective in reducing pain perception, evaluated by the numerical rating scale (NRS)^4^. However, discrete differences regarding the pattern of the analgesic profile after long-term stimulation of these targets are still under investigation. Here, we have shown that while subthalamic stimulation showed a significantly decrease in sensory complains related to parkinsonism, as previously demonstrated^6^, pallidal stimulation failed to significantly attenuate this phenomenon. On the other hand, both stimulated targets were able to attenuate bodily discomfort without interfering with pain not explained by other known conditions. Interestingly, the improvement in the overall motor score was not significantly correlated with the improvement of pain-related subscores. This may highlight the importance of investigating and treating pain in PD beyond motor improvement. Another significant difference between STN and GPi-DBS was the central expression of beta-endorphin, where pallidal stimulation actually decreased the expression of this pivotal opioid. Taken together, our data corroborate with other reports regarding the benefits of STN-DBS in “real-life” scales regarding pain^2–4^.

To better assess the different characteristics of PD-related pain, Pellaprat and coworkers investigated pain parameters using the bodily discomfort subscore of the PDQ-39 and item 17 of the UPDRS-II regarding sensory complaints related to parkinsonism^12^, as evaluated in our study. Similar to our findings, they demonstrated that STN-DBS decreased these scores and the McGill Pain Questionnaire score in the presence of early morning dystonia. Furthermore, we found a non-significant decrease of 50% in the subscore of NMSS “pain not explained by other known conditions” after STN-DBS. Only 4 out of 14 patients (28.5%) allocated to subthalamic stimulation did not show improvement on this subscore.

Regarding the cohort of patients subjected to pallidal stimulation, we found a non-significant decrease of 25% in the NMSS subscore “pain not explained by other conditions” and a significant decrease of 65% in the PDQ-30 subscore “bodily discomfort.” Seven out of 13 patients (53.8%) who underwent pallidal stimulation did not exhibit improvement in this subscore. No difference was found in the UPDRS-II subscore “sensory complaints related to parkinsonism.” It is important to consider that the conflicting data observed may be related to the etiology and nature of pain itself. While most of the data demonstrate that DBS fails to attenuate nociceptive, central, and neuropathic pain, it has also been shown that DBS significantly improves dystonic and musculoskeletal pain^13^. Taken together, these data highlight the importance of characterizing individuals with PD suffering from different types of pain in the attempt to provide optimal and individualized treatment.

Interestingly, individuals undergoing subthalamic stimulation also showed an increase of 43% in central beta-endorphin levels, whereas individuals undergoing GPi-DBS showed a significant decrease of 47%. In our small cohort of patients, the levels of beta-endorphin negatively correlated with subscores of “pain not explained by other conditions” and “bodily discomfort”. In other words, individuals with decreased levels of pain had higher levels of beta-endorphin in the CSF six months after DBS in both targets. Considering that both stimulation targets had a similar response in bodily discomfort and sensory complains related to parkinsonism, it is possible that beta-endorphin central expression is, at least partially, related to pain not explained by other conditions. The opioidergic system is often associated with analgesia. Individuals with persistent pain demonstrate decreased levels of beta-endorphin in the CSF^14^, while opioidergic deficits are also associated with PD^15,16^. However, although opioids can be used for pain associated with PD, they induce several adverse effects^17^. Hence, activating the opioidergic system using non-pharmacological approaches may be an optimal strategy to control pain in individuals with PD. This may explain why STN-DBS individuals demonstrated slightly better analgesic effects, considering that GPi-DBS was unable to increase opioid levels in the central nervous system. Of note, activation of the opioidergic system may also be accountable for other DBS effects in PD. In this sense, non-human primates showed attenuation of levodopa-induced dyskinesia after oral administration of mu-opioid agonists^18^. Nevertheless, the mechanisms by which DBS may modulate central beta-endorphin are still a matter of debate. In a pre-clinical design, Gee and coworkers have shown that subthalamic stimulation is able to attenuate the firing frequency of a subset of neurons in the periaqueductal grey matter (PAG)^18^. Considering the pivotal role of PAG-opioid projections in the analgesic pathway^19^, it is possible that DBS modulates beta-endorphin by modulating the firing response in the analgesic descending pathway. However, further studies are needed to better comprehend the effect of subthalamic and pallidal stimulation on analgesic-related structures.

In conclusion, we provide confirmation that both STN and GPi stimulation were able to decrease pain characteristics six months after continuous treatment. However, subthalamic, rather than pallidal stimulation, showed a discreet analgesic superiority, especially concerning sensory complains related to parkinsonism. This discreet superiority may be related to the discrete increase in central beta-endorphin levels. Considering patients where sensory complains, including pain, are a major concern that pharmacological treatment fails to attenuate, we recommend considering subthalamic stimulation as a reliable treatment. It is worth mentioning the limitations of the current study, where we discuss results derived from a single-center, open-label, ancillary trial with a limited sample size and follow-up of only six months. Furthermore, the participants of this trial were not stratified accordantly with different etiologies of pain, including dystonic pain, which is most often observed in PD. Nevertheless, neurochemical mediators involved in the stimulation response should be addressed in larger cohorts to provide a better understanding of DBS mechanisms, potential biomarkers for individualized treatment and brain target assignment, and closed-loops stimulation paradigms in the future.

## Methods

### Patients

This was an ancillary study from a prospective clinical trial approved by the Ethics Committee of Hospital Sírio-Libanês (CAAE:02857412.9.0000.5461) and registered at Clinical Trials (ClinicalTrial.gov) with the identifier NCT02647372. Twenty-seven patients with PD were randomly subjected to STN-DBS (*N* = 13) or GPi-DBS (*N* = 14). Patients were recruited from February 2013 to December 2014 at the Sao Paulo University Clinics. The inclusion criteria were PD patients diagnosed according to international criteria^20^ in a mid-stage phase with motor complications from prolonged levodopa treatment and DBS indication. The exclusion criteria were severe swallowing or speech disorders related to PD, anatomical abnormalities, pre-existing uncontrolled medical conditions, and surgical contraindications. The patients were prospectively evaluated before and six months after DBS. Written informed consent was obtained from all the patients included in this study. Participants were randomized, and clinicians received the prospective target of stimulation in sealed opaque envelopes. Participants were blinded to the surgery’s target, while. Clinicians were not blinded at any moment of the study.

### Outcomes

Patients were evaluated through the Non-Motor Symptoms Scale (NMSS), Unified Parkinson’s Disease Rating Scale (UPDRS) Part III, and Parkinson’s Disease Questionnaire (PDQ) at the preoperative examination and six months after surgical treatment. To better evaluate sensory complications due to PD, we analyzed the following separately: (i) *Item 27 from the NMSS*. This item rates pain not explained by other known conditions, where severity is rated as 0 = none, 1 = mild, 2 = moderate, and 3 = severe; and frequency is scored as 1 = rarely, 2 = often, 3 = frequent, and 4 = very frequent; (ii) *Item 17 from the UPDRS-III*. This item relates to sensory complaints related to parkinsonism, where 0 = none, 1 = occasional numbness, tingling, or mild aching, 2 = frequent numbness, tingling, or aching; not distressing, 3 = frequent painful sensations, and 4 = excruciating pain; (iii) *Bodily discomfort subscore from the PDQ-39*. This item concerns three different questions regarding painful muscular cramps or spasms, pain in the joints or body, and discomfort regarding cold or hot. These questions were rated as 0 = never, 1 = rarely, 2 = sometimes, 3 = frequently, and 4 = always.

### Evaluation of beta-endorphin

The patients underwent lumbar CSF collection before and six months after DBS treatment. The samples were quantified using Luminex xMAP (HNPMAG-35K, Merck Millipore). The assay was performed according to the manufacturer’s recommendations.

### Statistical analysis

Normality of distributions was evaluated using the Shapiro-Wilk test. Patient scales and central beta-endorphin expression were evaluated by the Wilcoxon matched-pairs test comparing pre- and post-surgery for the STN and GPi groups separately. Associations between two variables (beta-endorphin expression versus pain not explained by other known conditions and bodily discomfort) were evaluated using the Spearman correlation test. Statistical analyses were performed using GraphPad Prism software (San Diego, CA, version 5.0), and significance was considered when *p* < 0.05 for all evaluations.

### Reporting summary

Further information on research design is available in the Nature Research Reporting Summary linked to this article.

### Supplementary information

Reporting summary

## Data Availability

The datasets generated and/or analyzed during the current study are available from the corresponding author upon reasonable request from the REDCap database (https://redcap.iephsl.org.br). Restriction of data availability regards any information that may disrupt the anonymity of participants.
